# Positional‐based cloning ‘fail‐safe’ approach is overpowered by wheat chromosome structural variation

**DOI:** 10.1002/tpg2.20106

**Published:** 2021-07-01

**Authors:** Ella Taagen, James Tanaka, Alvina Gul, Mark E. Sorrells

**Affiliations:** ^1^ Plant Breeding and Genetics Section, School of Integrative Plant Science Cornell University Ithaca NY 14853 USA; ^2^ Atta‐ur‐Rahman School of Applied Biosciences National University of Sciences and Technology Islamabad Pakistan

## Abstract

Positional‐based cloning is a foundational method for understanding the genes and gene networks that control valuable agronomic traits such as grain yield components. In this study, we sought to positionally clone the causal genetic variant of a 1000‐grain weight (TGW) quantitative trait loci (QTL) on wheat (*Triticum aestivum* L.) chromosome arm 5AL. We developed heterogenous inbred families (HIFs) (>5,000 plants) for enhanced genotypic resolution and fine‐mapped the QTL to a 10‐Mbp region. The transcriptome of developing grains from positive and negative control HIF haplotypes revealed presence–absence chromosome arm 5AS structural variation and unexpectedly no differential expression of genes within the chromosome arm 5AL candidate region. Evaluation of genomic, transcriptomic, and phenotypic data, and predicted function of genes, identified that the 5AL QTL was the result of strong linkage disequilibrium (LD) with chromosome arm 5AS presence or absence (HIF *r* ^2^ = 0.91). Structural variation is common in wheat, and our results highlight that the redundant polyploid genome's masking of such variation is a significant barrier to positional cloning. We propose recommendations for more efficient and robust detection of structural variation, including transitioning from a single nucleotide polymorphism (SNP) to a haplotype‐based approach to identify positional cloning targets. We also present nine candidate genes for grain yield components based on chromosome arm 5AS presence or absence, which may unveil hidden variation of homoeolog dosage‐dependent genes across the group five chromosome short arms. Taken together, our discovery demonstrates the phenotypic resiliency of polyploid genomic structural variation and highlights a considerable challenge to routine positional cloning in wheat.

## Core Ideas

- Wheat chromosome arm presence–absence phenotypic resiliency confounded SNP‐based QTL mapping
- Polyploid genomic structural variation is common and a significant barrier to fine‐mapping
- Strategies to detect structural variation are essential for routine positional cloning in wheat
- Structural genomic variation may facilitate identification of homoeolog dosage‐dependent genes

**Abbreviations**
ARF: auxin response factor
BLUP: best linear unbiased prediction
DEG: differentially expressed gene
DPA: days postanthesis
GBS: genotyping‐by‐sequencing
GFD: grain‐fill duration
GL: grain length
GO: gene ontology
GW: grain width
HC: high confidence
HD: heading date
HIF: heterogenous inbred family
HT: plant height
IWGSC: International Wheat Genome Sequencing Consortium
KASP: Kompetitive allele specific polymerase chain reaction
LC: low confidence
LD: linkage disequilibrium
LOD: logarithm of odds
QTgw.cnl‐5A−: W7984 allele
QTgw.cnl‐5A+: Opata allele
QTL: quantitative trait loci
RIL: recombinant inbred line
RNA‐seq: RNA sequencing
SNP: single nucleotide polymorphism
SPS: spikelets per spike
SSR: simple sequence repeat
SynOpDH: W7984 × Opata M85 doubled‐haploid reference population
SynOpRIL: W7984 × Opata M85 recombinant inbred line
TGW: 1000‐grain weight
TILLING: target‐induced local lesions in genome

## 1 INTRODUCTION

Common wheat (*Triticum aestivum* L.) and durum wheat [*Triticum turgidum* L. subsp. *Durum* (Desf.) van Slageren] deliver more than 20% of the daily calories and protein consumed by the human population (http://www.fao.org/faostat/en/#data). To sustainably support the world's growing population and farm profitability, wheat productivity must increase under fewer production hectares. Despite wheat's crucial bearing on global food security, the genes and gene networks controlling wheat grain yield remain poorly understood. Grain yield is a highly polygenic trait that is influenced by genetic and environmental factors at every stage of plant growth (Slafer, 2003). An additional challenge to pinpointing the genetic control of grain yield is posed by the large polypoid wheat genome, where the phenotypic consequence of a single‐variable locus can be masked by the redundant copies of other homoeologous genomes (Borrill et al., 2018). Given the complexity of identifying genetic controls of grain yield, a reductionist approach that considers highly heritable yield components is a valuable strategy to improve our understanding of underlying genes and gene networks (Brinton & Uauy, 2018; Zhang et al., 2018a).

Total grain yield is a balancing act between yield components such as spikes per unit of area, grain number per spike, and grain weight. Quantitative trait loci for yield components have been identified on every wheat chromosome, but many of these QTL span broad genomic regions and offer limited impact for breeding. Some of the latest advances in wheat genomics, such as the advent of an annotated reference genome and gene editing techniques (Borrill et al., 2018), now allow us to go beyond QTL mapping and invest in positional cloning. Positional‐based cloning identifies a gene through fine‐mapping, sequencing, and functional validation. Fine‐mapping resolution is limited by the low frequency and uneven distribution of crossovers between linked genetic markers. A ‘fail‐safe’ approach is to develop heterogenous inbred families (HIFs) or near isogenic lines, which share a highly inbred and homogenous genome but are segregating for the genomic region of interest, for example, as a QTL (Brinton et al., 2017; Kuzay et al., 2019; Tuinstra et al., 1997). Continuous inbreeding of several thousand HIF progeny will produce distinct crossovers that delimit the QTL to a gene variant that can be confidently associated with the plant's phenotype, for example using target‐induced local lesions in genome (TILLING) populations or gene editing.

1 **Core Ideas**

- Wheat chromosome arm presence–absence phenotypic resiliency confounded SNP‐based QTL mapping
- Polyploid genomic structural variation is common and a significant barrier to fine‐mapping
- Strategies to detect structural variation are essential for routine positional cloning in wheat
- Structural genomic variation may facilitate identification of homoeolog dosage‐dependent genes

We set out to positionally clone the causal variant underlying a 1000‐grain weight (TGW) and grain morphology QTL on chromosome arm 5AL, *QTgw.cnl‐5A*, as previously identified in the Synthetic W7984 x Opata M85 spring wheat doubled‐haploid reference population (herein abbreviated ‘W7984’, ‘Opata’, and ‘SynOpDH’) (Breseghello & Sorrells, 2006; Sorrells et al., 2011; Williams et al., 2014). Strong associations between grain weight or morphology and markers in this region have also been reported (Brinton et al., 2017; Kato et al., 2000; Sukumaran et al., 2018). In this study we leveraged the SynOp recombinant inbred line (SynOpRIL) population for HIF development by identifying two founder F_6_ lines segregating for the *QTgw.cnl‐5A* flanking markers (*5A_283300187* and *5A_482369161*). We screened more than 5,000 progenies over five generations (2017–2019 field and greenhouses) and narrowed *QTgw.cnl‐5A* to a 10‐Mbp region flanking 37 high‐confidence (HC) genes. This fine‐mapping advancement coincided with the publication from Gutierrez‐Gonzalez et al. (2019) that identified the short arm of chromosome 5A was missing from the W7984 parent and prompted us to evaluate the implications for our positional cloning.

The W7984 chromosome arm deletion may not have been detected by the larger wheat community, or earlier in our research, in part because of no aberrant morphological variation or infertility, which is characteristic of whole‐chromosome arm deletions (Zhang et al., 2019, 2020). Here, we used a subset of both SynOpDH and SynOpRIL populations to (a) significantly and consistently map *QTgw.cnl‐5A* across four environments; (b) develop HIFs for fine‐mapping *QTgw.cnl‐5A* and disrupt linkage disequilibrium (LD) with chromosome arm 5AS presence (+) and absence (−); (c) conduct grain growth rate analysis for greater phenotypic resolution; (d) measure the transcriptome of chromosome arm 5AS+/*QTgw.cnl‐5A*+ and chromosome arm 5AS−/*QTgw.cnl‐5A−* HIFs; and (e) conduct gene ontology (GO) term enrichment analysis. Based on genetic data, phenotypic associations, early grain development expression profiles, and predicted function of genes, we suggest that *QTgw.cnl‐5A* is the result of strong LD with chromosome arm 5AS presence or absence (SynOpDH *r* ^2^ = 0.95, HIF *r* ^2^ = 0.91). The resources invested for positional cloning *QTgw.cnl‐5A* were overpowered by chromosomal structural variation, and we discuss the challenges that persist for identifying gene function in wheat.

We also present nine candidate genes on chromosome arm 5AS that may impact yield components including TGW, grain length (GL), grain width (GW), and spikelets per spike (SPS). These results lay the foundation for identifying hidden variation of homoeolog dosage‐dependent and functionally redundant genes on the group five chromosome short arms. Altogether, our findings highlight the phenotypic resiliency of polyploid genomic structural variation and present recommendations for future approaches to positional cloning.

## 2 MATERIALS AND METHODS

### 2.1 QTL validation plant materials

A synthetic hexaploid wheat, generated by crossing the durum wheat ‘Altar 84′ (AABB) with an *Aegilops tauschii* Coss. (DD) accession, crossed with the spring wheat ‘Opata 85′, were used to generate 215 doubled‐haploids (SynOpDH) via chromosome doubling, and 2,039 RILs (SynOpRIL) (Sorrells et al., 2011). Both populations segregate for the absence of W7984 chromosome arm 5AS, or presence of Opata, but the structural variation remains to be characterized in all 2,039 SynOpRILs (Supplemental file_S1.5.csv). A subset of each mapping population was used for this study. One hundred forty‐nine entries from SynOpDH, along with parental checks and two commercial checks ‘Glenn’ and ‘Tom’, were grown in two replicated and randomized 1‐m, single‐row plots in Ithaca, NY, during the field seasons (April–August) of 2016 (Caldwell field), 2017 (Caldwell field), and 2018 (Caldwell and Helfer field). An unbalanced set of 13 additional entry observations were included during best linear unbiased prediction (BLUP) phenotype calculations for 162 total entries. All field trials were nonirrigated. All of the spikes in each 1‐m row were hand harvested and threshed with a belt thresher (Almaco). Heading date (HD), TGW, GL, and GW were measured and used to validate the QTL across years and environments as well as identify the flanking marker positions of the QTL based on Chinese Spring genome assembly released by the International Wheat Genome Sequencing Consortium (IWGSC), henceforth RefSeq v1.0 (International Wheat Genome Sequencing Consortium (IWGSC), 2018). Gene annotations presented in this study are based on RefSeq v1.1 annotation. While RefSeq v2.0 assembly was available at the time of analysis, the annotation was still under development.

### 2.2 Development of HIF‐derived fine‐mapping population

The fine‐mapping population was constructed using HIFs derived from two F_6_ SynOpRIL founder entries (7‐956 and 7‐1201) heterozygous for markers flanking the QTL on chromosome arm 5AL (*5A_283300187* and *5A_482369161;* marker origin is RefSeq v1.0 and the syntax is chromosome_RefSeq v1.0 position). In order to increase the genetic resolution of the QTL and maintain an isogenic background genome, individual progeny from these two entries were inbred X generations (F_6:X_) and genotyped to screen for recombinants between the QTL flanking markers. Heterozygous entry advancement, recombinant evaluation, and progeny testing took place 2016–2019 until the F_6:4_ or F_6:5_ generation. Inbreeding cycled between Snyder or Caldwell fields in Ithaca, NY, and Cornell University Guterman greenhouse. Under field evaluation, 100 progenies of each heterozygous entry were advanced, and 20 progenies from a recombinant entry were advanced for validation. Greenhouse evaluation space was limited, and this environment was only used for recombinant validation testing between field cycles. The greenhouse environment was supplemented with artificial lighting to obtain a 16‐h day, 8‐h night photoperiod with 21–23 °C day and 15–17 °C night temperatures. Individual plant identity was tracked throughout HIF development and all of the spikes of each plant were hand harvested into a coin envelope and belt threshed. For example, HIF entry 7‐956‐2‐89‐2‐17‐01 started with F_6_ founder entry 7‐956 and out of the 100 progenies planted, the second plant was heterozygous for the QTL flanking markers and was selected from F_6:1_ planting, the 89th plant was selected from F_6:2_ planting, the second plant was selected from F_6:3_ planting, at F_6:4_ the 17th plant recombined between the QTL flanking markers and was validated at the F_6:5_ generation. Homozygous recombinant and sister entries, Opata (+) and W7984 (−) allele controls without a crossover between QTL flanking markers, were selected and evaluated for pre‐ and postharvest phenotypes in field experiments.

In total, more than 5,000 progenies were screened for recombinants between the QTL flanking markers and 109 (F_6:4_ or F_6:5_) recombinant haplotypes were selected for the fine‐mapping population. In addition, nine sister entries with Opata alleles and 11 with W7984 alleles spanning *QTgw.cnl‐5A* (+/– controls) were selected. The 129 fine‐mapping population entries were genotyped with 31 Kompetitive allele specific polymerase chain reaction (KASP) markers spanning *QTgw.cnl‐5A*. One hundred twenty‐nine entries, along with parental checks and two commercial checks, Glenn and Tom, were grown in two replicated and randomized 1‐m, single‐row plots in Ithaca, NY, during the field seasons of 2019 (Snyder field) and 2020 (Caldwell and Helfer field). All of the spikes in each 1‐m row were hand harvested and threshed with a belt thresher. Heading date, plant height (HT), grain‐fill duration (GFD), SPS, TGW, GL, and GW were measured and used for *t* test comparisons between recombinant haplotypes and control haplotypes to narrow the QTL flanking markers (Supplemental file_S2.7.csv).

### 2.3 Phenotypic data and statistical analysis

The preharvest phenotypes measured in this study include HD, HT, days postanthesis (DPA) grain morphology, and GFD; postharvest include SPS, TGW, GL, and GW. The HD was recorded as Julian date of 50% spike emergence. Plant height was measured as the average height (cM) from the ground to spike tip in a 1‐m, single‐row plot. Days postanthesis was recorded by tagging individual spikes at 0 DPA, when anthers at the center spikelet are light green and pollination can be confirmed within 24‐h of tagging. The GFD was recorded as the time between 0 DPA and physiological maturity (when the peduncle turned yellow) for a 1‐m, single‐row plot.

The spikes from every plot in this study were hand harvested with sickles or scissors. The SPS was recorded as the average spikelet number from 10 random spikes in a plot. The GL and GW were measured using >150 grains per sample on a WinSEEDLE STD4800 system flatbed scanner. The number of grains was recorded and then weighed as a proxy for TGW. This process was replicated without resampling for a given plot, and the average GL, GW, and TGW was recorded. Phenotypes for the SynOpDH across four field–year environments and the fine‐mapping population across three field–year environments can be found in Supplemental file_S1.1.csv and file_S2.1.csv, respectively.

In order to evaluate *QTgw.cnl‐5A+/−* haplotypes and phenotypes across field and year combinations, univariate linear models with random genotype and environment effects and correlated information (HD, chromosome arm 5AS+/−, or HIF RIL founder) fixed effects were fitted with the *R/lme4* package (Supplemental file_S1.8.xlsx / script_S1.md and file_S2.9.csv / script_S2.md) (Bates et al., 2015; R Core Team, 2020). Models were evaluated based on their broad‐sense heritability and Akaike information criterion. Fixed‐effect significance was assessed with *R/car* package Wald chi‐square test (Fox & Sanford, 2019). Broad‐sense heritability (*H* ^2^) estimates for HD, TGW, GL, and GW in the SynOpDH population were calculated using the following equation:

$$H^{2}=\frac{\sigma_{G}^{2}}{\sigma_{G}^{2}+\frac{\sigma_{G\times E}^{2}}{l}+\frac{\sigma_{E}^{2}}{rl}}$$

where $\sigma_{G}^{2}$ is the genetic variance, $\sigma_{G\times E}^{2}$ is the G×E variance, $\sigma_{E}^{2}$ is the residual variance, *r* is the number of replications, and *l* is the number of environments. Best linear unbiased predictions were obtained from the univariate linear models for SynOpDH and HIF phenotypes. The SynOpDH BLUP phenotypes were used for QTL mapping with the *R/qtl* package (Broman et al., 2003). The HIF BLUP phenotypes were used for Welch two‐sample *t* tests with control and recombinant haplotypes and chromosome arm 5AS+/− haplotypes (Supplemental file_S2.7.csv, script_S2.md) (R Core Team, 2020).

For the grain growth rate analysis, GL, GW, fresh weight, and dry weight were measured at 0, 4, 10, 16, and 22 DPA in a replicated subset of 10 fine‐mapping haplotypes (five *QTgw.cnl‐5A−* and five *QTgw.cnl‐5A+*; Supplemental file_S3.1.csv) during the 2019 field season. The experiment was replicated in the Cornell University Guterman greenhouse during spring 2020 for four HIF haplotypes (same entries as RNA sequencing [RNA‐seq] experiment) and also included measurements at 28 DPA and senescence (Supplemental file_S3.2.csv). For every haplotype and replicate, 10 primary spikes were used per timepoint. From each of the 10 spikes, 10 primary developing grains in florets 1 and 2 were taken only from the central spikelets and the average GL, GW, fresh weight, and dry weight (5 d in a 30 °C dryer) were measured. Single‐trait mixed models with a fixed interaction between haplotype and DPA and a random effect of plot (field) or tube (greenhouse) nested within haplotype were fitted with the *R/lme4* package (Bates et al., 2015). Post hoc comparison of least‐squares means for haplotype and DPA was performed within each model using the *R/emmeans* package in addition to multiple‐test correction *P* values (Supplemental script_S3.md) (Lenth et al., 2019).

### 2.4 Genetic map construction and QTL mapping

A genetic map for the 162‐entry SynOpDH subset was constructed with 1,551 polymorphic genotyping‐by‐sequencing (GBS) and simple sequence repeat (SSR) markers that were previously published and using the R package ‘qtl’ (Broman et al., 2003; Poland et al., 2012; Sorrells et al., 2011). The function ‘estmap’ was used to estimate the genetic distances using the ‘kosambi’ mapping function followed by maximum‐likelihood analysis of marker order on each chromosome using the function ‘ripple’. Any missing genotypes were imputed with the function ‘fill.geno’ and ‘imp’ method. The QTL were identified by a single QTL model genome scan using the function ‘scanone’ with the Haley–Knott regression method. A .05 significance logarithm of odds (LOD) threshold for each phenotype was determined with the function ‘scanone’ and ‘n.perm = 1000′. Next, the percentage variance explained by each significant QTL was calculated. The RefSeq v1.0 physical position of flanking markers was determined for each significant QTL as well. Later a consensus marker for chromosome arm 5AS presence or absence was added for QTL mapping. The code for the genetic map and QTL mapping can be found in Supplemental script_S1.md.

### 2.5 High‐resolution *QTgw.cnl‐5A* genetic mapping

Seedling leaf tissue samples for DNA extraction were collected in the field or greenhouse from a single plant in each plot or pot with two replicates of the parents per 96‐well plate. The DNA was extracted from lyophilized leaf tissue using a modified cetyl trimethylammonium bromide extraction (Doyle & Doyle, 1990). The KASP assays were developed to screen recombinant entries and their progeny as well as to identify sister entries. The KASP markers were generated from polymorphisms identified using the wheat exome‐capture and regulatory‐capture sequence of parental entries of the Wheat‐CAP project (https://www.triticeaecap.org/wheatcap‐germplasm‐list/) (Gardiner et al., 2019; He et al., 2019a ). We also used the 10+ Genome Project data repository to BLAST our KASP marker sequences and confirmed genome and chromosome specificity and the marker order across Chinese Spring and eleven diverse wheat cultivars: ArinaLrFor, Jagger, Julius, Lancer, Landmark, Mace, Norin61, Stanley, SY‐Mattis, Zavitan, and Spelt (Supplemental file_S2.6.csv, script_S2.md) (Walkowiak et al., 2020). The KASP assay procedure followed the methods outlined in Makhoul et al. (2020) including PACE‐IR Genotyping Master Mix with a low ROX level and thermo‐cycling conditions according to 3CR bioscience protocols. On each SNP reaction plate, the parent lines, check lines, and at least one water sample were included as controls. All experiments were repeated at least twice. If clear genotyping clusters were not obtained, the KASP marker was abandoned. The clustering patterns of the three KASP markers that span *QTgw.cnl‐5A* (10‐Mbp region) are available as Supplemental file_2.13.pdf. *QTgw.cnl‐5A* flanking KASP markers (*5A_283300187* and *5A_482369161*) were used to screen the HIF progeny, and all 31 KASP markers were used to select the fine‐mapping population (129 entries). Later, the fine‐mapping population was genotyped with three SSR markers for chromosome arm 5AS presence or absence (Supplemental file_S2.5.csv). The homogeneous genetic background of the HIFs and the high heritability of the traits allowed us to differentiate their association with *QTgw.cnl‐5A* from the causal effects of chromosome arm 5AS structural variation.

### 2.6 Gene expression

We used RNA‐seq to compare the levels of expression of genes within the candidate *QTgw.cnl‐5A* region across chromosome arm 5AS and to evaluate the HIF isogenic background genome. The plant tissue was sampled from developing grains at 4 and 8 DPA from four HIF haplotypes: 7‐956‐2‐19‐1‐31‐03 (Opata control), 7‐956‐2‐19‐1‐44 (W7984 control), 7‐956‐2‐19‐1‐31‐05 (recombinant I), and 7‐956‐2‐12‐1‐69‐07 (recombinant II). The four haplotypes were grown in a completely randomized design in the greenhouse during spring 2020. We sampled 500 mg of whole grain tissue per biological replicate. At the 4‐DPA timepoint, 40 primary spikes were collected for each haplotype and 10 developing primary grains from the central spikelets of 10 randomly sampled spikes (four biological replicates, 100 hundred grains per biological replicate) were immediately frozen in liquid nitrogen and stored at −80 °C. The process was repeated at the 8‐DPA timepoint but only two randomly sampled spikes per biological replicate were necessary because of the rapidly growing grains (four biological replicates, 20 grains per biological replicate). The tissue was ground with liquid nitrogen and mortar and pestle and total RNA was extracted using a modified hot borate protocol (Wan & Wilkins, 1994). Quantity and quality of the isolated total RNA was determined using a Biotek Epoch 2 Spectrophotometer with Nanodrop functionality and gel electrophoresis. Three of the four biological replicates were sent to Novogene for further quality screening and nondirectional 150‐bp paired‐end reads mRNA sequencing (four haplotypes, three biological replicates at two timepoints: 24 samples). The raw sequence reads were submitted to the SRA of NCBI under BioProject ID: PRJNA693003.

The bioinformatics pipeline and scripts for gene expression analysis can be found in Supplemental script_S4.md. Paired‐end reads from raw sequence data were imported to the Cornell University BioHPC server and all computational analysis was performed within the command line and R. Imported reads quality was checked with ‘FastQC’ (Andrews, 2010). The IWGSC RefSeq v1.0 assembly and RefSeq v1.1 high and low confidence gene annotations were downloaded from https://urgi.versailles.inra.fr/download/iwgsc/IWGSC_RefSeq_Assemblies/v1.0/andhttps://urgi.versailles.inra.fr/download/iwgsc/IWGSC_RefSeq_Annotations/v1.1/. The genome was indexed, and the sequence reads were aligned with STAR for both low‐confidence (LC) and HC RefSeq v1.1 gene annotation (Dobin et al., 2013). Batch effects were accounted for using the ‘ComBat_seq’ function in the *R/sva* package, and differential expression between haplotypes was analyzed with the *R/DESeq2* package (Leek et al., 2020; Love et al., 2014). A false discovery rate cut‐off value of 0.01 and log2 fold‐change threshold of two was used to select the list of differentially expressed genes at 4 and 8 DPA between the haplotype contrasts. Variance stabilizing transformation of the DESeq2 adjusted counts was used for principal component analysis and heatmaps of the count matrix. The SNP variants in the *QTgw.cnl‐5A* region were identified with the SAMtools BCFtools command ‘mpileup’ (Li et al., 2009).

### 2.7 Gene ontology term enrichment

At this point we excluded LC gene models from further analysis. The list of 556 HC genes differentially expressed between all three positive haplotypes (Opata control, recombinant I, and recombinant II) and the negative haplotype (W7984 control) at 4 and 8 DPA can be found in Supplemental file_S4.20.csv. We obtained RefSeq v1.1 HC gene annotation GO terms from the Wheat@URGI JBrowse portal (Alaux et al., 2018). In total, we extracted GO terms for 93,141 genes, which included all 556 differentially expressed genes (DEGs). The 93,141 genes served as the universe of genes for a GO term enrichment study with the R package ‘GOseq’ to test overrepresentation of GO terms among the DEGs (Young et al., 2010). We considered over‐represented GO terms with Benjamini–Hochberg false discovery rate adjusted *P* value < .05 to be significant.

We also identified DEGs with GO terms known to be associated with spike architecture and early grain development including cell proliferation, division, growth, and differentiation; meristem initiation, maintenance, and transition; flowering time; floral organ development; mitosis; regulation of gene expression; DNA methylation and gene silencing; auxin; cytokinin; response to stress; phosphorylation; photosynthesis; nucleosome assembly; starch; nucleic acid metabolic process; protein metabolic process; glucose and sucrose; ubiquitin pathway; brassinosteroid; microtubule; and endoreduplication (Brinton & Uauy, 2018; Li et al., 2018; Wang et al., 2017). Arabidopsis [*Arabidopsis thaliana* (L.) Heynh.] and rice (*Oryza* sativa L.) orthologs were identified using *Ensembl*Plants (https://plants.ensembl.org/index.html), arabidopsis.org TAIR Gene Search, and funricegenes (Yao et al., 2018). Additional packages required to reproduce results or figures include *R/tidyverse*, *R/kintr*, *R/kableExtra*, *R/gt*, *R/rstatix*, *R/rlang*, *R/ggpub*r, *R/vsn*, *R/PCAtools*, *R/pheatmap*, *R/reshape*, *R/ggbio*, *R/biovizBase*, *R/gghighlight*, *R/inauguration* (Blighe & Lun, 2020; Henry et al., 2020; Huber et al., 2002; Iannone et al., 2020; Kassambara, 2020a, 2020b; Kolde, 2019; Wickham, 2018; Wickham et al., 2019; Xie, 2020; Yin et al., 2012, 2020; Yutani, 2020; Zhu, 2020; Bedford‐Petersen, 2021).

## 3 RESULTS

### 3.1 A QTL on chromosome 5A is associated with increased grain weight

A genetic map was developed for the SynOpDH population comprising of 1,551 polymorphic molecular markers (Supplemental file_S1.9.csv, script_S1.md). Using the SynOpDH phenotype BLUP values, calculated from four field–year observations, significant QTL for TGW were identified on chromosomes 2D (*QTgw.cnl‐2D*), 5A (*QTgw.cnl‐5A*), and 6A (*QTgw.cnl‐6A*). *QTgw.cnl‐2D* and *QTgw.cnl‐6A* colocalized with QTL for GL, and *QTgw.cnl‐5A* colocalized with a QTL for GW. A QTL for HD on the long arm of chromosome 5A was also detected, spanning the *VRN‐1* gene (Yan et al., 2003). The flanking and peak markers, LOD score, positions in the genetic map and wheat reference genome, and grain weight percentage variation explained by each QTL are described in Supplemental script_S1.md. A univariate linear model for TGW with random entry and environment effects and fixed‐interaction effects between peak markers of the three TGW QTL showed highly significant effects for all three QTL but no significant interactions (Supplemental script_S1.md). These results indicated that *QTgw.cnl‐5A*, the focus of this study, could be mapped regardless of combinations with *QTgw.cnl‐2D* and *QTgw.cnl‐6A*.

**FIGURE 1:**
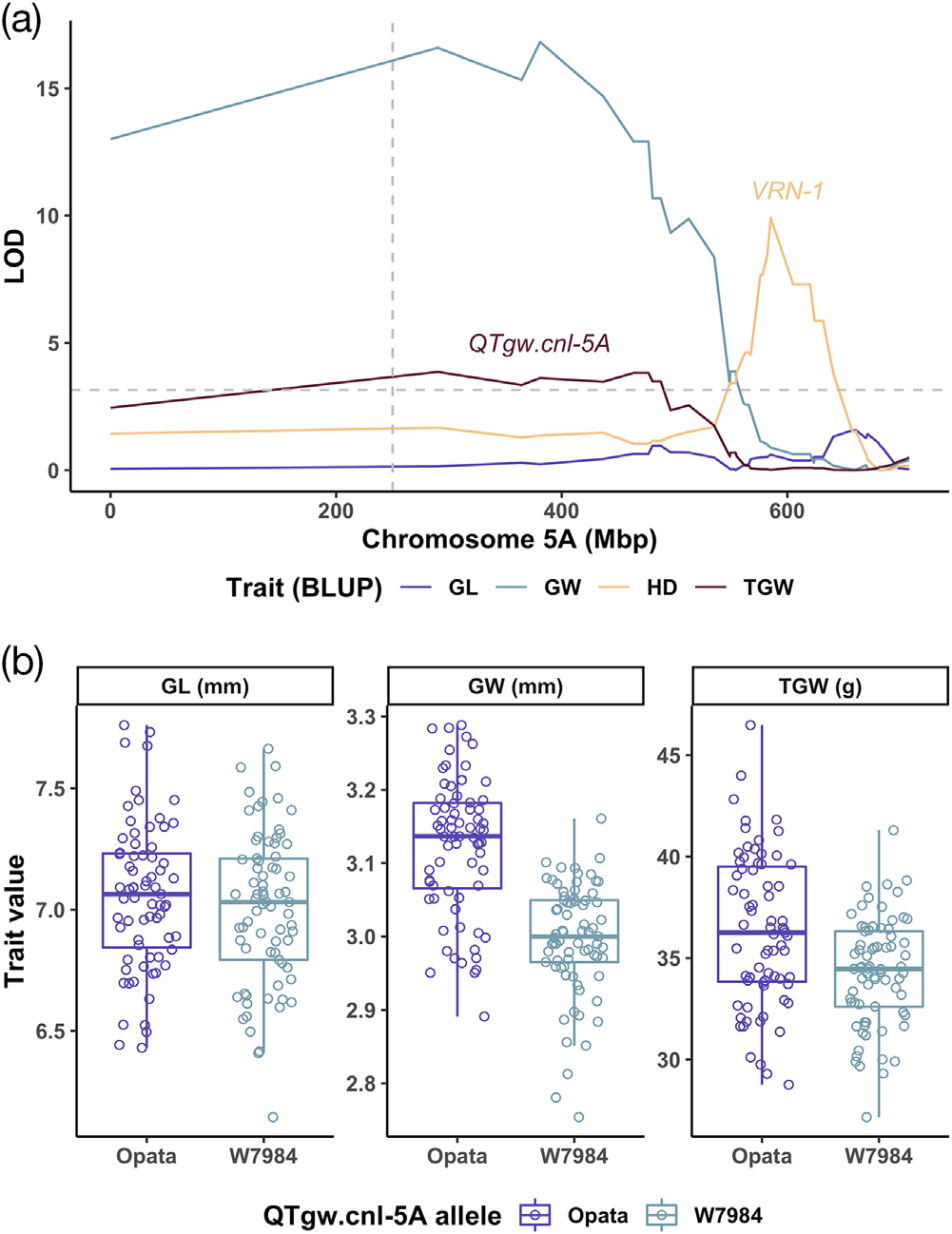
(a) Quantitative trait loci detected for 1000‐grain weight (TGW), grain width (GW), and heading date (HD) in the SynOpDH population on chromosome 5A. A single marker was used to represent the chromosome arm 5AS structural variation. Values on the *x* axis represent RefSeq v1.0 physical position, and the vertical dashed line represents the centromere. The dashed line on the *y* axis represents the logarithm of odds (LOD) significance threshold. (b) Best linear unbiased prediction (BLUP) phenotype distribution for SynOpDH entries subset by *QTgw.cnl‐5A* allele, based on flanking marker genotypes

The *QTgw.cnl‐5A* flanking markers were *wmc705* (0.1 cM, LOD 3.86) and *synopGBS284* (4.49 cM, LOD 3.49) and accounted for 10.39% of the grain weight and 37.9% of the width variation. Upon inspection of the flanking marker RefSeq v1.0 physical positions (*wmc705*, 290 Mbp and *synopGBS284*, 487 Mbp), it became clear that *QTgw.cnl‐5A* mapped to chromosome arm 5AL and the genetic map lacked markers on chromosome arm 5AS. Our QTL mapping occurred before Gutierrez‐Gonzalez et al. (2019) published dense GBS linkage maps that indicated that the SynOpDH population segregated for the presence or absence of the short arm on chromosome 5A. Later, we adjusted the marker order to reflect physical positions and incorporated a single marker for QTL mapping that represented the chromosome arm 5AS structural variation (Figure 1a).

Phenotypic distributions and ANOVA tests of the SynOpDH population revealed Opata provides the increased TGW and GW allele (Figure 1b, Table 1). We hypothesized that the increased grain weight is mediated by differences in GW rather than GL, which are under independent genetic control (Gegas et al., 2010). The broad‐sense heritability for TGW, GL, GW, and HD were 0.68, 0.75, 0.81, and 0.78 respectively. SynOpDH entries with the *QTgw.cnl‐5A* Opata allele (+) were, on average, 6.4% heavier and 4.2% wider than the W7984 allele (−) and significantly different across all environments (Table 1).

**TABLE 1:** Mean 1000‐grain weight (TGW), grain length (GL), grain width (GW), and heading date (HD) of SynOpDH entries. Percentages (%) refer to the trait value gained in SynOpDH QTgw.cnl5A+ (Opata allele) entries vs. QTgw.cnl5A− (W7984 allele) entries. The QTgw.cnl5A+ and QTgw.cnl5A− allele categories were determined by genotype at the peak quantitative trait loci marker 5A_341510829. Broad‐sense heritability (*H* ^2^) considered trait observations across all locations. Replicates grown in locations with spatial variation are reported independently and denoted with a letter in parentheses. Asterisks indicate significance determined by ANOVA for each location or best linear unbiased prediction (BLUP)

Year	Location	Genotype	TGW	GL	GW	HD
			g	mm		Julian date
2016	Caldwell	*QTgw.cnl‐5A+*	39.19	7.16	3.26	–
		*QTgw.cnl‐5A−*	36.49	7.09	3.08	–
Trait value gained (%)			7.4***	0.98ns^†^	5.7***	–
2017	Caldwell	*QTgw.cnl‐5A+*	–	6.58	2.84	–
		*QTgw.cnl‐5A−*	–	6.53	2.68	–
Trait value gained (%)			–	0.81ns	6.1***	–
2018	Caldwell (A)	*QTgw.cnl‐5A+*	40.36	7.25	3.26	184.71
		*QTgw.cnl‐5A−*	38.37	7.28	3.14	182.64
Trait value gained (%)			5.2**	−0.39ns	3.7***	1.1*
2018	Caldwell (B)	*QTgw.cnl‐5A+*	39.97	7.24	3.26	185.2
		*QTgw.cnl‐5A−*	37.8	7.25	3.13	182.89
Trait value gained (%)			5.7**	−0.21ns	4.04***	1.3**
2018	Helfer (A)	*QTgw.cnl‐5A+*	31.04	7.08	3.08	187.05
		*QTgw.cnl‐5A−*	28.55	6.97	2.92	184.8
Trait value gained (%)			8.7***	1.70ns	5.4***	1.2*
2018	Helfer (B)	*QTgw.cnl‐5A+*	31.61	7.03	3.09	186.91
		*QTgw.cnl‐5A−*	29.77	6.97	2.96	184.79
Trait value gained (%)			6.2**	0.81ns	4.5***	1.1*
BLUP		*QTgw.cnl‐5A+*	36.46	7.07	3.12	186.02
		*QTgw.cnl‐5A−*	34.26	7.004	2.996	183.77
Trait value gained (%)			6.4***	0.92ns	4.2***	1.2**
*H* ^2^			0.68	0.75	0.81	0.78

^*^Significant at the .05 probability level. ^**^Significant at the .01 probability level. ^***^Significant at the .001 probability level.

^†^ns, nonsignificant.

### 3.2 HIFs differing for *QTgw.cnl‐5A* show a 21.3% difference in TGW

To further investigate the effect of *QTgw.cnl‐5A* on TGW, HIF populations were generated from two F_6_ SynOpRIL entries (7‐956 and 7‐1201) heterozygous for flanking KASP markers *5A_283300187* and *5A_482369161*. The fine‐mapping population selection was based on recombination between flanking markers and sister line genotypes, as individual plant phenotypes for TGW were too variable. Our fine‐mapping population development occurred before results by Gutierrez‐Gonzalez et al. (2019) were published.

In 2016, we grew a seed increase of the SynOpRIL population (F_6:1_) and identified HIF founder lines 7‐956 and 7‐1201, heterozygous for *QTgw.cnl‐5A* flanking markers. We screened 600 F_6:1_ progeny (F_6:2_) during the 2017 field season (Snyder field) with *QTgw.cnl‐5A* flanking KASP markers and identified no crossovers within the maker interval and 45 plants that remained heterozygous. Nine hundred progenies from the F_6:2_ heterozygous plants (F_6:3_) were evaluated in a greenhouse during the winter of 2017, and 44 plants with crossovers within the flanking KASP interval, seven W7984 sister lines (recombination outside of flanking KASP interval), and six Opata sister lines were identified. Five additional KASP markers were developed across the target region based on SNPs identified in parental exome‐capture data (He et al., 2019a). The F_6:4_ generation, validated during the 2018 field season (Caldwell field), consisted of inbreeding 1,620 entries with a single heterozygous flanking marker and progeny tests of the 44 recombinants. We identified 65 new homozygous recombinant plants within the flanking KASP interval, four W7984 sister lines, and three Opata sister lines. An additional 24 KASP markers were developed between the flanking markers based on SNPs identified in the exome‐capture and regulatory‐capture data (Gardiner et al., 2019). Progeny tests of the 65 recombinant plants (F_6:5_) as well as replicates of the 44 F_6:4_ validated recombinants, and 20 sister lines were planted in a greenhouse during winter 2018 and characterized with the full set of 31 KASP markers. We screened an additional 1,514 segregating F_6:6_ plants during the 2019 field season (Snyder) but did not find a crossover event that reduced the target region. The 109 recombinants and 20 sister lines were selected for the fine‐mapping population and field‐based phenotypic evaluation.

**FIGURE 2:**
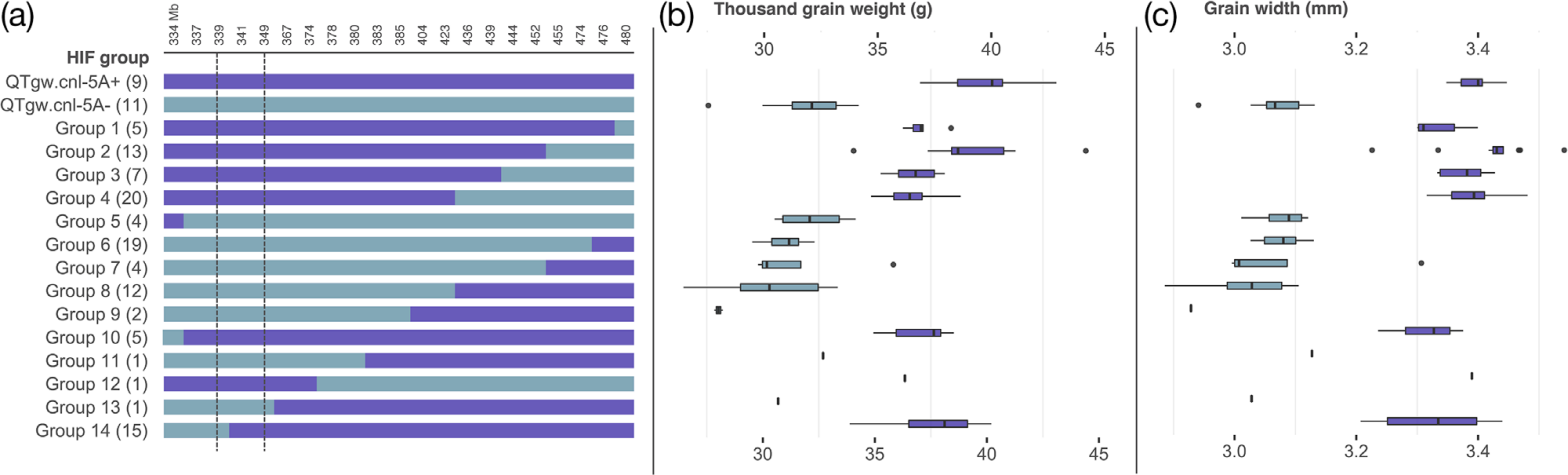
Fine‐mapping of heterogenous inbred family (HIF) recombinant entries and trait variation pinpoints *QTgw.cnl‐5A* to a 10‐Mbp interval on wheat chromosome arm 5AL. (a) Graphical genotypes of 129 HIFs grouped by shared recombination intervals (KASP marker positions not to scale, purple Opata SNPs, blue W7984 SNPs). Number of HIFs in each group noted within parentheses. (b, c) Best linear unbiased prediction (BLUP) phenotype boxplot distribution of each HIF group. Boxes are colored based on Opata‐ or W7984‐like phenotype determined by *t* test (Supplemental script_S2.md)

The fine‐mapping population was grown in 2019 (Snyder) and 2020 (Caldwell and Helfer) and evaluated for HD, HT, GFD, SPS, TGW, GL, and GW (Table 2). Significant differences in TGW and GW associated with the peak marker *5A_341510829* narrowed the *QTgw.cnl‐5A* candidate region to a 10‐Mbp interval containing 37 HC and 84 LC genes flanked by markers *5A_339757917* and *5A_349628635* (Figure 2; Supplemental script_S2.md). The Opata allele frequency at SNP *5A_341510829* was 0.58, and the W7984 allele frequency was 0.42. The lower variability of the HIFs and homogenous background genome increased the resolution of additional yield component quantitative traits, including significant associations for GL, SPS, and HT with *QTgw.cnl‐5A* (Table 2). There was no significant difference in HD or GFD, suggesting the difference in grain weight may be due to the grain filling rate rather than duration. Given the additional phenotypes associated with the candidate gene region, we decided to explore the transcriptome of *QTgw.cnl‐5A*+ and *QTgw.cnl‐5A*− haplotypes.

**TABLE 2:** Mean heading date (HD), grain‐fill duration (GFD), plant height (HT), spikelets per spike (SPS), 1000‐grain weight (TGW), grain length (GL), and grain width (GW) of SynOp HIF entries. Percentages (%) refer to the trait value gained in SynOp HIF QTgw.cnl5A+ (Opata allele) entries vs. QTgw.cnl5A− (W7984 allele) entries. The QTgw.cnl5A+ and QTgw.cnl5A− allele categories were determined by genotype at the peak QTL marker, 5A_341510829. Replicates grown in locations with spatial variation are reported independently denoted with a letter in parentheses. Asterisks indicate significance determined by ANOVA for each observation or best linear unbiased prediction (BLUP)

Year	Location	Genotype	HD	GFD	HT	SPS	TGW	GL	GW
			Julian date	d	cM		g	mm	
2019	Snyder (A)	QTgw.cnl‐5A+	172.19	30.9	77.55	15.03	37.49	6.54	3.24
		QTgw.cnl‐5A−	172.57	30.98	74.96	14.76	31.22	6.4	2.93
Trait value gained (%)			−0.22ns	−0.26ns	0.35ns	1.80ns	20.08***	2.2***	10.4***
2019	Snyder (B)	QTgw.cnl‐5A+	171.59	30.4	81.99	15.11	38.69	6.58	3.28
		QTgw.cnl‐5A−	172	30.82	78.67	14.57	31.87	6.41	2.95
Trait value gained (%)			−0.24ns	−1.40ns	4.20ns	3.7**	21.4***	2.7***	11.1***
2019	Snyder (C)	QTgw.cnl‐5A+	173.56	–	83.81	15.59	38.33	6.45	3.24
		–	171.67	–	79.83	14.9	32.58	6.53	2.95
Trait value gained (%)			1.10ns	–	4.90ns	4.60ns	17.6***	−1.20ns	9.9***
2020	Caldwell	QTgw.cnl‐5A+	174.21	29.36	66.24	12.71	38.21	6.92	3.53
		QTgw.cnl‐5A−	173.81	29.46	62.94	12.43	31.41	6.81	3.21
Trait value gained (%)			0.23ns	−0.35ns	5.2*	2.20ns	21.7***	1.6**	10.2***
2020	Helfer	QTgw.cnl‐5A+	172.13	29.23	58.73	11.71	36.58	6.8	3.45
		QTgw.cnl‐5A−	171.93	28.91	55.59	11.43	29	6.67	3.11
Trait value gained (%)			0.12ns	1.10ns	5.7**	2.40ns	26.1***	1.9***	11.02***
BLUP		QTgw.cnl‐5A+	172.57	29.32	71.2	13.62	37.72	6.71	3.37
		QTgw.cnl‐5A−	172.63	29.39	68.45	13.43	31.09	6.58	3.06
Trait value gained (%)			−0.04ns	−0.26ns	4.02**	1.4***	21.3***	1.99***	10.3***

*Significant at the .05 probability level. **Significant at the .01 probability level. ***Significant at the .001 probability level.

^†^ns, nonsignificant.

### 3.3 HIF variation in grain weight and morphology was significantly associated with early grain development

**FIGURE 3:**
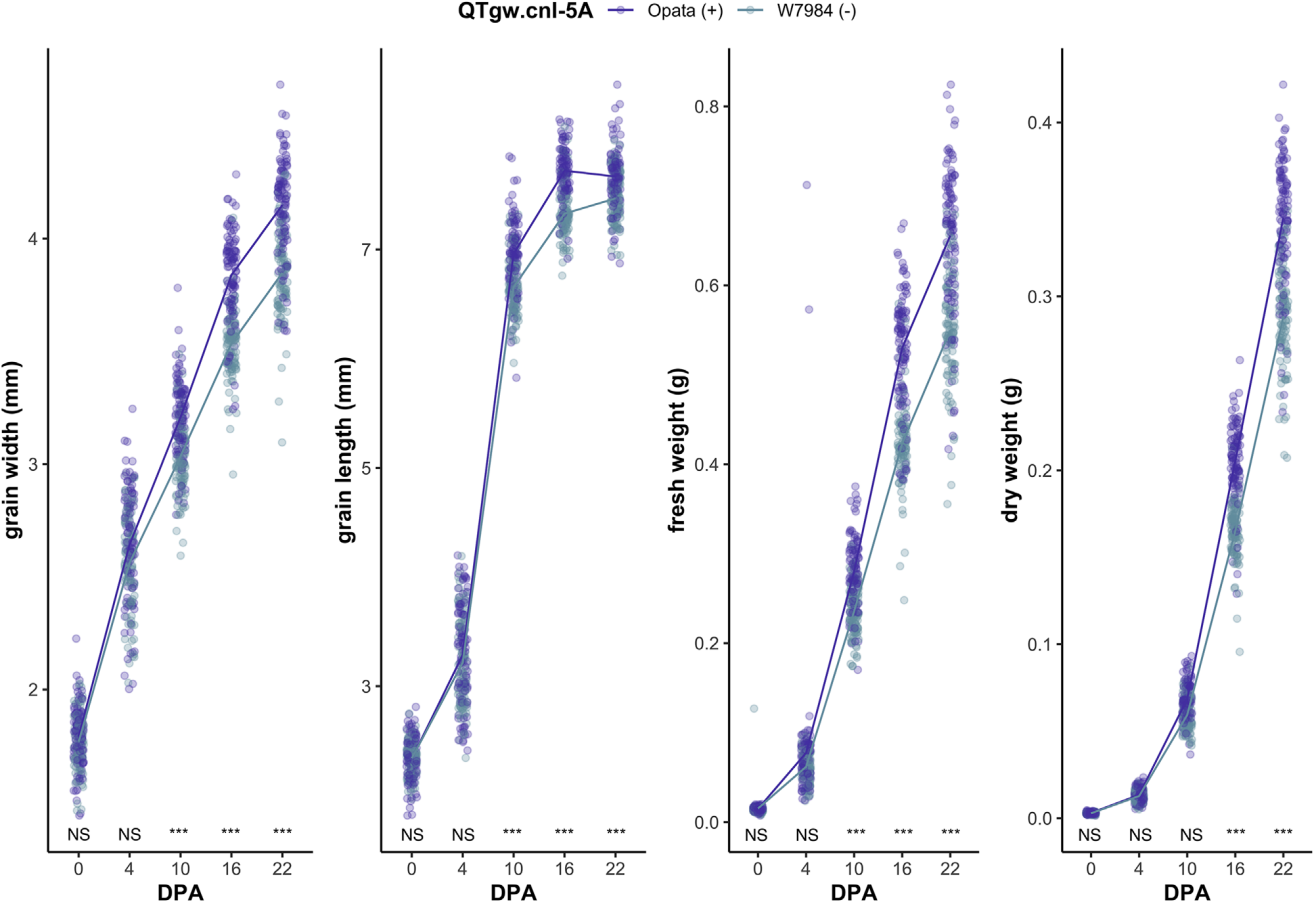
Developing grain phenotypes measured across 22 d postanthesis (DPA) for five *QTgw.cnl‐5A+* (purple) and five *QTgw.cnl‐5A−* (blue) heterogenous inbred families. Samples taken at 0, 4, 10, 16, and 22 DPA during 2019 field season. NS, nonsignificant; ***; *P* < .001

In order to better understand the mechanism driving the grain weight and morphology phenotype and to identify the optimum timepoints for our transcriptome study, we conducted a DPA analysis of the developing grains in five *QTgw.cnl‐5A*+ and five *QTgw.cnl‐5A*− HIF haplotypes. Grains of 10 replicated haplotypes were sampled from the 2019 field season at 0, 4, 10, 16, and 22 DPA. The first significant difference in GL, GW, and fresh weight was measured at 10 DPA, with *QTgw.cnl‐5A*+ grains 4.6% (*P* < .001) longer, 5.7% (*P* < .001) wider, and 18.9% (*P* < .001) heavier than *QTgw.cnl‐5A*‐ grains (Figure 3). The first significant difference in dry weight was measured at 16 DPA, with *QTgw.cnl‐5A*+ grains 25.2% (*P* < .001) heavier than *QTgw.cnl‐5A*‐ grains (Figure 3). These effects increased and were maintained at 22 DPA and senescence (data not shown).

The experimental design was repeated in a greenhouse environment and included additional measurements at 28 DPA and senescence with four HIF entries that would be selected for RNA‐seq (Opata control, W7984 control, recombinant I, recombinant II). The trend remained that the first significant difference in GW and fresh weight was measured at 10 DPA and dry weight at 16 DPA for Opata control vs. W7984 control grains. However, for comparisons between recombinant I and W7984 control and recombinant II and W7984 control, the difference for any phenotype was first measurable at 16 DPA (Supplemental script_S3.md). Differences in grain development between the field and greenhouse could be due to the growing environment, time of year, HIF haplotype, or too few experimental entry comparisons.

Grain development begins after pollination starting with rapid proliferation of cells that form the outer layer of the grain followed by the endosperm's cell division and expansion at ∼6 DPA (Brinton & Uauy, 2018; Li & Li, 2016). Our DPA study had consistent association with phenotypic variation at 10–16 DPA and, in conjunction with the 2019 and 2020 field data that found no significant difference in GFD among *QTgw.cnl‐5A*+ and *QTgw.cnl‐5A*− HIFs, suggests the difference in GW and grain weight is driven by the rapid endosperm cell division and expansion during early grain development.

### 3.4 *QTgw.cnl‐5A* in LD with chromosome arm 5AS structural variation

**FIGURE 4:**
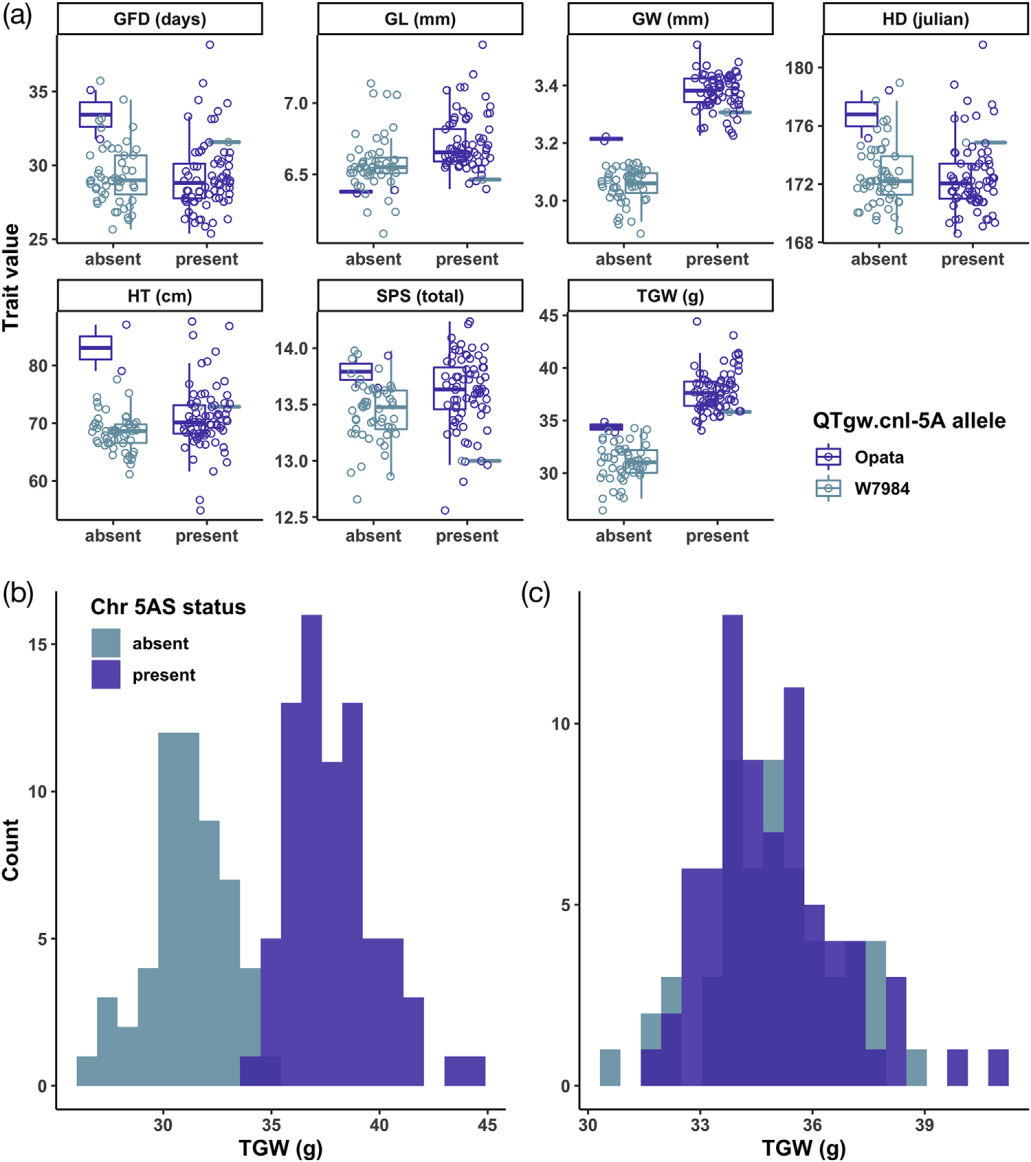
(a) Best linear unbiased prediction (BLUP) phenotype boxplot distribution for heterogenous inbred family (HIF) entries subset by chromosome arm 5AS presence or absence and colored by *QTgw.cnl‐5A* allele (KASP *5A_341510829*; Opata, purple; W7984, blue). Of the 129 fine‐mapping entries, 73 had haplotype chromosome short arm 5A+ / *QTgw.cnl‐5A*+, 53 had haplotype chromosome short arm 5A− / *QTgw.cnl‐5A*−, one had haplotype chromosome short arm 5A+ / *QTgw.cnl‐5A*−, and two had haplotype chromosome short arm 5A− / *QTgw.cnl‐5A*+. (b) Original BLUP 1000‐grain weight (TGW) histogram, subset by chromosome arm 5AS presence (purple) or absence (blue). (c) Chromosome arm 5AS structural variant fixed effect BLUP TGW histogram, subset by presence (purple) or absence (blue). GFD, grain‐fill duration; GL, grain length; GW, grain width; HD, heading date; HT, plant height; SPS, spikelets per spike

Upon learning from Gutierrez‐Gonzalez et al. (2019) that the SynOpDH population was segregating for the presence or absence of the short arm on chromosome 5A, we genotyped our fine‐mapping population with three SSR markers for chromosome arm 5AS presence or absence. The frequency of chromosome arm 5AS presence was 0.57 and absence was 0.43. Of the 129 fine‐mapping entries, 126 had either haplotype chromosome short arm 5A+/*QTgw.cnl‐5A*+ or chromosome short arm 5A−/*QTgw.cnl‐5A*−, with only one entry chromosome short arm 5A+/*QTgw.cnl‐5A*− and two entries chromosome short arm 5A−/*QTgw.cnl‐5A*+. The skewed haplotype distributions revealed chromosome arm 5AS structural variation and *QTgw.cnl‐5A* alleles are in very strong LD, with a correlation coefficient of 0.91. We tested for an interaction between the two loci using a univariate linear model for TGW with entry as a random effect and a fixed interaction between chromosome arm 5AS and peak marker *5A_341510829*. A Wald chi‐square test showed highly significant effects for chromosome arm 5AS and *5A_341510829* but no significant interaction. Phenotypic distributions of the fine‐mapping population indicated that trait values of the three recombinant HIFs that broke the LD (chromosome short arm 5A+/*QTgw.cnl‐5A*−, *n* = 1, and chromosome short arm 5A−/*QTgw.cnl‐5A*+, *n* = 2) are associated with the chromosome arm 5AS structural variant rather than *QTgw.cnl‐5A* allele (Figure 4a). In the context of Figure 2, the entry with chromosome short arm 5A+/*QTgw.cnl‐5A*− recombination belongs to group 7 and the two entries with chromosome short arm 5A−/*QTgw.cnl‐5A*+ recombination belong to group 14 (Supplemental file_S2.12.xlsx). Finally, we included the chromosome arm 5AS structural variant as a fixed effect in the univariate models for calculating HIF phenotype BLUPs. The new BLUP phenotypic distributions for HIF entries showed no significant difference for presence or absence of chromosome arm 5AS or the *5A_341510829* allele, suggesting the phenotypic variation was largely explained by the chromosome arm 5AS structural variation, and *QTgw.cnl‐5A* is a result of linkage (Figure 4b and 4c; Supplemental script_S2.md).

### 3.5 RNA‐seq of *QTgw.cnl‐5A* HIFs confirmed the significance of chromosome arm 5AS structural variation

We used RNA‐seq to investigate three questions with four HIF haplotypes (Table 3): (a) are genes in the *QTgw.cnl‐5A* candidate region differentially expressed? (b) are there genes on chromosome arm 5AS that are differentially expressed? and (c) do the HIFs share an isogenic background genome? We hypothesized that gene expression changes influencing phenotypic differences occurred before the first significant difference in grain morphology was detectable at 10 DPA and sampled whole grains at 4 and 8 DPA. We measured over 621 million reads across the 24 samples, with individual sample reads ranging from 21.8 to 37 million and an average of 25.8 million reads (SE = 0.7 million) per sample (Supplemental file_S4.02.csv, file_S4.03.csv). We aligned the reads to RefSeq v1.1 HC and LC annotations independently, and, on average, across samples 89.4 ± 0.6% of reads aligned to the HC gene annotation, and 89 ± 0.62% aligned to the LC gene annotation (for read counts see Supplemental file_S4.md).

**TABLE 3:** Heterogenous inbred family (HIF) haplotypes selected for RNA sequencing. Chromosome arm 5AS structural variation (chr 5AS), QTgw.cnl‐5A genotype (marker 5A_341510829) where Opata is (+) and W7984 is (−), 1000‐grain weight (TGW), grain width (GW)

HIF^a^	NAME	CHR 5AS	*QTgw.cnl‐5a*	TGW	GW
				g	mm
7‐956‐2‐19‐1‐31‐03	Opata control	Present	+	37.895	3.408
7‐956‐2‐19‐1‐44	W7984 control	Absent	−	29.983	3.091
7‐956‐2‐19‐1‐31‐05	Recombinant I	Present	+	36.341	3.39
7‐956‐2‐12‐1‐69‐07	Recombinant II	Present	+	40.199	3.44

^a^ HIF entries were chosen to prioritize isogenic lineage and QTL resolution over chr 5AS and *QTgw.cnl‐5A* recombinants.

**FIGURE 5:**
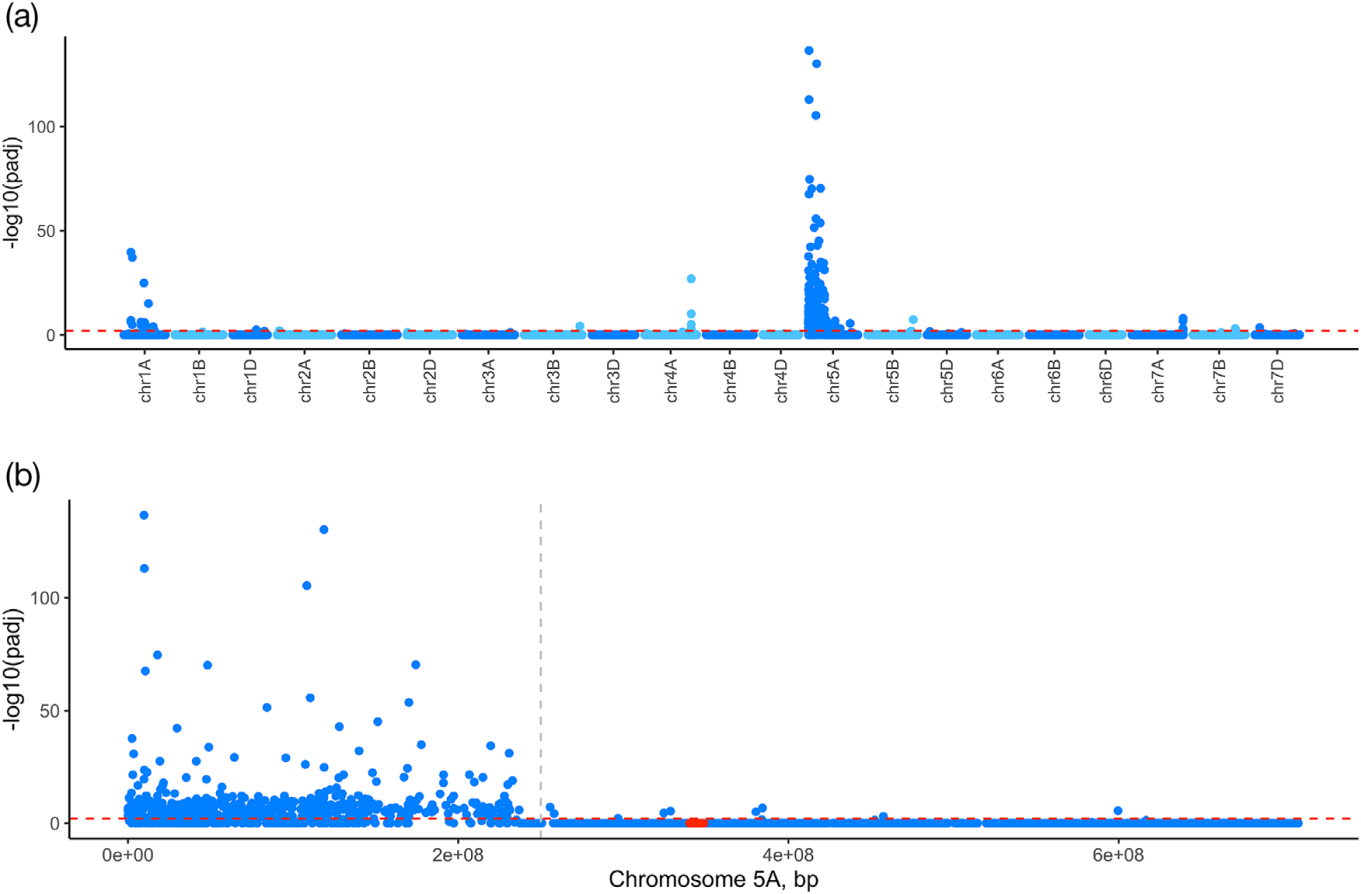
Differentially expressed genes (a) genome wide and (b) on chromosome 5A, for Opata vs. W7984 control haplotypes at 8 d postanthesis (DPA). Each point represents a gene, ordered by RefSeq v1.0 physical position along the *x* axis. The *y* axis represents the differential expression significance, −log base 10 adjusted *P* value. The red dashed line marks *P* value = .01. In (b) the grey dashed line represents the centromere, and the red points highlight genes between *QTgw.cnl‐5A* flanking KASP markers *5A_339757917* and *5A_349628635*

We performed differential expression comparisons between the HIFs at both timepoints. Independent comparison between HIFs for Opata control, recombinant I, and recombinant II vs. W7984 control did not identify any DEGs at 4 and 8 DPA in the *QTgw.cnl‐5A* candidate region (chromosome 5A *339757917*–*349628635 bp*) for the HC or LC gene annotation. However, there were 532 significant DEGs in common among these HIF comparisons at 4 DPA, 469 at 8 DPA, and 556 in total at either timepoint for the HC gene annotation. Of the 556 DEGs, 15 were on chromosome 1A, one on 1B, one on 3A, 532 on 5A short arm, three on 5A long arm, one on 5D, two on 7A, and one on 7B. The 535 genes on chromosome 5A were all differentially expressed because of no expression in the W7984 control HIF. None of the DEGs homoeologous copies were differentially expressed. Given only 21 of the 556 DEGs were on chromosomes other than chromosome 5A, the differential expression comparisons between HIFs also validated the fine‐mapping population's isogenic background genome (Figure 5; Supplemental script_S4.md).

These results prompted us to evaluate the considerable resources we had invested in an otherwise reliable positional cloning approach for *QTgw.cnl‐5A*. It was possible we sampled tissue at the wrong timepoints or that *QTgw.cnl‐5A* association with grain weight and morphology was driven by a posttranscriptional or posttranslational modification. We investigated the 10‐Mbp candidate region of the 24 samples’ raw reads for SNP variants from the reference genome and identified only one variant. Among the three W7984 control biological replicates, a 3′ (T → C) was present in *TraesCS5A02G160900* (Supplemental file_S4.18.xlsx). This gene codes for the third largest subunit of RNA polymerase II and the SNP, termed BA00617686, had previously been identified by the CerealsDB Axiom 820K and 35K SNP Array (Winfield et al., 2016). The SNP has not been associated with any phenotype, and there is no literature on the Arabidopsis and rice orthologs that implicate the gene in grain development.

Centromeres are vital for proper chromosomal segregation during mitosis and meiosis. Consequentially, experimentally derived chromosome arm deletion lines in wheat often lack a clean break at the centromere (Gill, 1996). The centromere of chromosome 5A is near 250 Mbp in Chinese Spring RefSeq v1.0. Although Gutierrez‐Gonzalez et al. (2019) reported that the entire short arm was missing for W7984 chromosome 5A, our transcriptome study revealed 15 genes expressed in the W7984 control haplotype at 237–250 Mbp. None of the W7984 haplotype expressed genes were differentially expressed with the positive haplotypes, which made them unlikely candidates for the observed phenotypic variation. The centromeric position can change among cultivars, but this has not previously been associated with a structural variation event (Walkowiak et al., 2020). We propose that W7984 short arm of chromosome 5A broke approximately 13 Mbp from the centromere, as compared with Chinese Spring. This remains to be validated with a transcriptome study across additional SynOp entries and parents.

Across the W7984 haplotype samples, there were 23 genes that were unexpectedly expressed on chromosome arm 5AS (0–237 Mbp) (Supplemental file_S4.14.xlsx). We investigated the read counts of these genes and identified 11 of the genes were differentially expressed, but still from less expression by W7984 samples. We explored the 23 gene sequences using *EnsemblPlants* and IWGSC BLAST, and, on average, there was 94.6 ± 1.7% sequence alignment with at least one homoeolog or paralog. On average, reads aligned to multiple loci represent 5.16 ± 0.22% of the total alignment (for read counts see Supplemental file_S4.md). We suggest these 23 genes have multiple sequence alignments and that their expression in the W7984 haplotype samples was incorrect.

Collectively, these findings indicate our approach to positional cloning was overpowered by chromosome arm 5AS structural variation and that *QTgw.cnl‐5A* was a result of strong LD. The presence or absence of chromosome arm 5AS was significantly associated with the yield component variation we identified in the fine‐mapping population. An in‐depth comparison of the 556 DEGs and association with yield components follows in the next section.

### 3.6 Chromosome arm 5AS differential expression provided insight into candidate genes

The phenotypes measured in the fine‐mapping population were chosen based on the quantitative phenotypic variation associated with *QTgw.cnl‐5A* in the SynOpDH background. In an isogenic fine‐mapping background, the phenotypic resolution was enhanced and included significant associations between HT, SPS, TGW, GL, and GW and the presence or absence of chromosome arm 5AS. The percentage difference between the *QTgw.cnl‐5A* +/‐ HIFs for these traits is reflective of additive, rather than dominance variation (Table 2). There were no other variable phenological or morphological traits among SynOpDH and HIF populations that were obvious during field trials. It is well known that wheat harbors hidden variation as a result of polyploidy and there are likely nonadditive and functionally redundant genes among the DEGs on chromosome 5A.

To better understand the DEGs, we performed GO enrichment analysis with GOsEquation (Young et al., 2010). The DEGs were associated with 1,919 out of 9,709 unique GO terms from the RefSeq v1.1. After statistical overrepresentation tests and correction for multiple testing, the only significant term was “killing of cells of other organism” (GO:0031640) (Supplemental script_S4.md). There were nine DEGs with GO:0031640, including the physical cluster of genes *TraesCS5A02G018000*, *TraesCS5A02G018800*, *TraesCS5A02G019000*, and *TraesCS5A02G019100*, which are orthologs of Arabidopsis bacterium and fungus response gene Osmotin‐like protein, OSM34 (Capelli et al., 1997). *TraesCS5A02G077600* is within the interval of a fusarium head blight resistance QTL, termed *Qfhs.ifa‐5AS*, and is orthologous to the rice Osmotin‐like protein Os12g0569300 (National Center for Biotechnology Information, 2021; Steiner et al., 2019). *TraesCS5A02G059000* is orthologous to the rice defense response gene OsPR1b, which is constitutively expressed at background levels (Luan & Zhou, 2015). Although the physical gene cluster of *TraesCS5A02G046300*, *TraesCS5A02G046400*, and *TraesCS5A02G046500* has no known orthologs in Arabidopsis or rice, they share the additional GO terms defense response to fungus (GO:0050832), binding (GO:0005488), peptidase activity (GO:0008233), extracellular region (GO:0005576), cell wall (GO:0005618), integral component of membrane (GO:0016021), cellular component organization (GO:0016043), and cellular metabolic process (GO:0044237).

The relative lack of GO term enrichment among chromosome arm 5AS+ and 5AS− HIF DEGs coupled with phenological variation characteristic of a single gene rather than entire chromosome arm, highlights the challenge of identifying hidden variation in a redundant polyploid genome. Despite the limited GO term enrichment, the positive association of SPS, TGW, GL, and GW with chromosome arm 5AS presence prompted us to investigate genes with GO terms related to spike architecture and early grain development. It was unclear if the difference in height was attributable to any DEGs measured at 4 or 8 DPA. There were 292 DEGs that had at least one GO term related to biological, cellular, and developmental processes during spike and early grain growth (Supplemental file_S4.16.csv). Of these genes, 52 were only differentially expressed at 4 DPA, and 10 genes were only differentially expressed at 8 DPA. The majority of the 230 genes that were differentially expressed at both timepoints decreased in expression across time. Only seven of the genes were on chromosomes other than 5A and are poor candidates based on GO terms and Arabidopsis and rice orthologs. We explored the Arabidopsis and rice orthologs for all 292 genes and present nine genes as candidates for wheat yield components in Table 4.

**TABLE 4:** Candidate *Triticum aestivum* RefSeq v1.1 genes and *Arabidopsis thaliana* and *Oryza sativa* orthologs. RefSeq v1.0 and v2.0 assembly chromosome

*T. aestivum*	RefSeq v1.0	Refseq v2.0	4‐DPA *P* value^a^	8‐DPA *P* value^a^	Selected GO terms^b^	*A. thaliana*	*O. sativa*
*TraesCS1A02G103900*	1AS	5AS	3.99 × 10^−8^	1.07 × 10^−7^	Regulation of cell size (GO:0008361)	*HAIKU2 (IKU2)*, regulator of seed size	*HAIKU2*
					Plant‐type cell wall organization (GO:0009664)		
					Multidimensional cell growth (GO:0009825)		
					Epidermal cell differentiation (GO:0009913)		
*TraesCS5A02G025900*	5AS	5AS	5.59 × 10^−30^	1.34 × 10^−17^	Post‐embryonic development (GO:0009791)	*YAB2*, abaxial cell fate	*OsYAB6*
					Cell differentiation (GO:0030154)		
					Reproductive structure development (GO:0048608)		
*TraesCS5A02G030300*	5AS	5AS	2.25 × 10^−9^	1.7 × 10^−7^	Brassinosteroid mediated signaling pathway (GO:0009742)	*BSL2* and *BSL3*, brassinosteroid‐insensitive suppressor family	*OsPPKL3*, negative regulator of grain length
*TraesCS5A02G037100*	5AS	5AS	4.99 × 10^−4^	–	Stamen development (GO:0048443)	*PID*, establishment of bilateral symmetry	*BIF2*, organogenesis during inflorescence
					Regulation of cell size (GO:0008361)		
					Cell projection (GO:0042995)		
					Auxin‐activated signaling pathway (GO:0009734)		
*TraesCS5A02G038300*	5AS	5AS	1.43 × 10^−16^	5.75 × 10^−10^	Auxin‐activated signaling pathway (GO:0009734)	*ARF6*, regulator of flower development and maturation	*OsARF25*, regulator of grain size
					Flower development (GO:0009908)		
*TraesCS5A02G103800*	5AS	5AS	6.54 × 10^−11^	7.61 × 10^−7^	Auxin metabolic process (GO:0009850)	*RGLG1* and *RGLG2*, regulator of apical dominance	–
					Cytokinin metabolic process (GO:0009690)		
*TraesCS5A02G106400*	5AS	5AS	4.69 × 10^−12^	4.93 × 10^−7^	Diacylglycerol kinase activity (GO:0004143)	*DGK2*, pollen and seed development	–
					Intracellular anatomical structure (GO:0005622)		
*TraesCS5A02G107800*	5AS	5AS	1.37 × 10^−14^	9.33 × 10^−14^	Response to ethylene (GO:0009723)	*VTC2* and *VTC5*, ascorbate biosynthesis	*OsGGP*, biomass production
					Response to auxin (GO:0009733)		
*TraesCS5A02G110600*	5AS	5AS	1.07 × 10^−13^	4.18 × 10^−9^	Phragmoplast (GO:0009524)	*AUG6*, mitotic and meiotic cell division	*Os02g0329300*, AUGMIN subunit 6
					Spindle assembly (GO:0051225)		

^a^ GOSeq2 adjusted *P* value from Opata vs. W7984 heterogenous inbred family haplotype comparisons, 4 and 8 d postanthesis (DPA). All haplotype comparisons can be found in Supplemental script_S4.md.

^b^ All GO terms are available in Supplemental file file_S4.17.xlsx

While the genes in Table 4 have not previously been associated with wheat yield components, their Arabidopsis and rice orthologs have been shown to regulate inflorescence development or seed and grain size. Based on RefSeq v1.0 alignment and v1.1 annotation, there are eight candidate genes on chromosome arm 5AS and one candidate gene on chromosome arm 1AS, *TraesCS1A02G103900*. *TraesCS1A02G103900* had GO terms related to cell size, cell growth, and epidermal cell differentiation and was orthologous to HAIKU2 (IKU2), a regulator of endosperm proliferation and cellularization (Li & Li, 2016; Luo et al., 2005). IKU2 loss‐of‐function is associated with reduced seed size in Arabidopsis (Luo et al., 2005). Notably, the homoeologous copies of *TraesCS1A02G103900, TraesCS5B02G012000*, and *TraesCS5D02G019400* map to the group 5 chromosomes not group 1. The homoeologs, as reported on *EnsemblPlants*, are orthologues to IKU2 as well. Our transcriptome analysis identified three other DEGs on chromosome 1AS clustered near *TraesCS1A02G103900* (9.76–9.98 Mbp), which also have homoeologs on the group 5 chromosome (Supplemental file_S4.19.xlsx). All four of these genes were differentially expressed as a result of zero read counts from the W7984 haplotype, while the other 11 DEGs identified on chromosome 1A have a mix of expression profiles among the four haplotypes. We submitted the *TraesCS1A02G103900* coding sequence to IWGSC BLAST RefSeq v1.0 and RefSeq v2.0, which identified 100% alignment with RefSeq v1.0 chromosome 1A and 71% with 5A and 100% alignment with RefSeq v2.0 chromosome 5A and 75% with 1A. We also identified 99% alignment with chromosome 5A of the durum wheat (‘Svevo’) genome assembly v1. Given the haplotype expression profile, homoeologous copies on group 5 chromosomes and RefSeq v2.0 and Durum alignment, we believe *TraesCS1A02G103900* was misannotated by RefSeq v1.1 and belongs on chromosome arm 5AS. The eight additional candidate genes on chromosome arm 5AS and their ortholog functions are discussed in the proceeding section.

## 4 DISCUSSION

In this study, we discovered that a stable and robust QTL was confounded by linkage with chromosome structural variation. We confirmed that the SynOpRIL population, in addition to the SynOpDH population described by Gutierrez‐Gonzalez et al. (2019) is segregating for presence of the Opata parent chromosome arm 5AS and absence of the majority of the W7984 parent chromosome arm 5AS. Furthermore, we associated the chromosome arm 5AS structural variation in an isogenic background with yield component phenotypes, characterized the early grain development transcriptome, and propose nine candidate genes for agronomically valuable traits on chromosome arm 5AS. Genomic structural variations are common across polyploids, and this study contributes to our understanding of the complexities associated with fine‐mapping in a polyploid species and discusses a more robust approach to positional cloning (Saxena et al., 2014; Song et al., 1995).

### 4.1 Detecting the chromosome structural variation

SynOpDH and SynOpRIL were two of the most widely referenced mapping populations leading up to the advent of the annotated wheat reference genome. Aside from the phenotypic variation subtleties, why was the W7984 chromosome arm deletion not detected sooner by the wider wheat community? The original W7984 × Opata crosses were developed in the early 1990s and were later reconstructed and expanded in 2011 (Sorrells et al., 2011). The rereleased population was genotyped with SSR and DArT markers, which are scored for the presence or absence of genomic fragments and would not have alerted researchers to the segregation of chromosome arm 5AS. When the SynOpDH high‐density GBS marker genetic map was developed in 2012 there was no reference genome available to flag that none of the markers mapped to chromosome arm 5AS (Poland et al., 2012). Our research group first noticed an anomaly on chromosome arm 5AS when we were unable to identify genome and chromosome arm specific polymorphic sites among the parent line exome‐capture and regulatory‐capture sequence for KASP marker development. In 2019, the dense GBS linkage maps created by Gutierrez‐Gonzalez et al. (2019) confirmed the significant structural variation of chromosome arm 5AS. It is also worth noting that many SynOp studies relied on a subset of either mapping population and may not have had a high enough frequency of the missing arm to detect the abnormality or were not focused on chromosome 5A. We urge previous studies involving SynOp populations and group 5 chromosomes to consider implications of chromosome arm 5AS structural variation on their results and recommend a list of SSR markers for any future studies that need to characterize SynOp entries not included in our current study (Supplemental file_S2.11.csv).

### 4.2 Why did *QTgw.cnl‐5A* map to chromosome arm 5AL?

Fundamentally, our positional cloning difficulties began with a QTL that mapped to the wrong chromosome arm. Our SynOpDH QTL map significantly associated markers on chromosome arm 5AL with grain weight and morphology across four environments, which is consistent with previous publications but inconsistent with our fine‐mapping results (Breseghello & Sorrells, 2006; Williams et al., 2014). Even when we were prompted by the findings of Gutierrez‐Gonzalez et al. (2019) to add a chromosome arm 5AS structural variation marker to the genetic map, *QTgw.cnl‐5A* still presented on the long arm. Potentially, R/qtl is insensitive to chromosome arm 5AS presence or absence because the *QTgw.cnl‐5A* causal variant is very near the centromere or part of the W7984 chromosome arm 5AS 13‐Mbp segment, but none of the genes in this region were differentially expressed. The lack of phenotypic resolution for quantitative traits like TGW and GW in the SynOpDH background may have also reduced our power to detect the association with chromosome arm 5AS, as seen among the minimal DH chromosome arm 5AS and *QTgw.cnl‐5A* haplotype recombinants (Supplemental script_S1.md). Alternatively, the genetic map of GBS and SSR markers on chromosome arm 5AL may not be genome specific, and false recombination events placed *QTgw.cnl‐5A* on chromosome arm 5AL. Finally, as we observed across *QTgw.cnl‐2D, QTgw.cnl‐5A*, and *QTgw.cnl‐6A*, GL and GW can independently influence grain weight. Although the GW 5A QTL spanned the centromere, because of its overlap with *QTgw.cnl‐5A* we considered the difference in GW and TGW to be a pleiotropic effect. Collectively, these results encouraged us to develop a fine‐mapping population targeting *QTgw.cnl‐5A* on chromosome arm 5AL.

### 4.3 Candidate genes for yield components

Grain yield is a key economic driver behind the success of wheat production and the value chain. However, grain yield is determined at the end of plant growth and is directly or indirectly impacted by all genes. Balancing the mechanisms driving the source tissue growth, the number of grains produced by a plant, and the weight of those grains are a key challenge for breeders as the constituent yield components compete for resources. Notably, in this study, we have identified a positive combination among five traits (HT, SPS, TGW, GL, and GW) under independent genetic control on Opata chromosome arm 5AS. Phenotypic variation associated with chromosome arm 5AS structural variation, significant differential expression during early grain development, GO terms, and orthologs were leveraged to identify nine candidate genes that may impact spike or grain development (Table 4). Ultimately, the genomic resolution of this study was limited by presence–absence structural variation and it is premature to conclude that any of the nine candidate genes contribute to the observed spikelet and grain morphology variation. Independent knockout studies of each candidate gene in HIF entries with Opata chromosome arm 5AS are necessary for functional validation. A discussion of each candidate gene follows.

Among the DEGs we identified *TraesCS5A02G025900*, an ortholog to Arabidopsis gene *YAB2* and rice gene *YAB6*, which regulate abaxial cell fate in leaf, sepal, petal, stamen, and carpel primordia (Siegfried, 1999). In Arabidopsis, *YAB2* acts redundantly with the larger YABBY gene family, and single loss‐of‐function plants exhibited no measurable organ polarity defects (Stahle et al., 2009). There are 21 YABBY genes subdivided into six families in wheat, but functional validation studies and phenotypic associations remain unexplored (Buttar et al., 2020). Given the functional redundancy identified in Arabidopsis, and large gene family in wheat, the loss of *TraesCS5A02G025900* (TaYABBY5‐5A) in our study was likely masked by gene family copies.

Another candidate gene that may impact wheat spike inflorescence development is *TraesCS5A02G037100*. *TraesCS5A02G037100* is orthologous to Arabidopsis gene *PID*, and rice and maize (*Zea mays* L.) gene *BIF2*, which are involved in inflorescence organogenesis (Benjamins et al., 2001; He et al., 2019b; McSteen et al., 2007). While loss‐of‐function plants in maize produced fewer spikelets and florets, rice did not suffer from flower initiation defects and indicated *BIF2* function is likely veiled by redundant partners (He et al., 2019b). *TraesCS5A02G037100* has not been associated with SPS in wheat outside of our chromosome arm 5AS structural variation study.

*TraesCS5A02G103800* is orthologous to Arabidopsis RING domain Ligase genes *RGLG1* and *RGLG2*, which modulate the directional flow of auxin (Yin et al., 2007). While single mutants in either gene have no apparent phenotypic effect, double‐mutant plants exhibit loss of apical dominance and altered phyllotaxy. *TraesCS5A02G103800* and homoeologous gene copies may harbor hidden variation regulating spike development in wheat.

*TraesCS1A02G103900* is orthologous to *IKU2*, a gene involved in endosperm growth and seed development signaling pathways (Luo et al., 2005). The RefSeq v1.1 annotation maps *TraesCS1A02G103900* to chromosome 1A; however, our results indicate this gene was misannotated and maps to chromosome 5A. *SHB1, IKU1, IKU2*, and *MINI3* function in the same signaling pathway to control seed size in Arabidopsis, and loss of any gene can reduce seed size (Li & Li, 2016). Loss of the *IKU2* wheat ortholog impact on grain size remains to be functionally validated outside of our chromosome arm 5AS structural variation study.

*TraesCS5A02G030300* is orthologous to protein phosphatases with Kelch‐like domains genes *BSL2* & *BSL3* in Arabidopsis, and *OsPPKL3* in rice (Kim et al., 2018). Protein phosphatases with Kelch‐like domains genes are known positive effectors of brassinosteroid signaling in plants, and brassinosteroid has been shown to regulate seed size and shape in Arabidopsis (Jiang et al., 2013). *OsPPKL3* and its homolog *OsPPKL1* are negative regulators of GL, but rare allelic variation is associated with extra‐large grains and increased yield (Zhang et al., 2012). The wheat ortholog to *OsPPKL1*, *TaGL3‐5A*, is associated with longer GL and has been functionally validated, suggesting *TraesCS5A02G030300* may be a strong candidate for exploring allelic diversity and association mapping (Yang et al., 2019).

Another potential grain size regulator is *TraesCS5A02G038300*, or *TaARF14*, which is orthologous to auxin response factor (ARF) *ARF6* in Arabidopsis and *OsARF25* in rice (Nagpal et al., 2005; Xu et al., 2020; Zhang et al., 2018b). Dosage effects among ARF knockouts in Arabidopsis found that *ARF6* impacts the timing of flower maturation. *OsARF25* has been functionally validated in an auxin signaling pathway where it binds to the promoter of *OsERF142*, a positive regulator of cell expansion and ultimately GL (Zhang et al., 2018b). A recent comprehensive atlas of wheat ARF gene expression suggests *TaARF14* is required to promote stamen development, but functional validation outside of chromosome arm 5AS structural variation remains to be determined.

*TraesCS5A02G106400*, *TaDGK5A*, is the wheat ortholog of the Arabidopsis gene *DGK2*, a diacylglycerol kinase essential for reproductive organ development (Angkawijaya et al., 2020). There are seven DGK genes in Arabidopsis, which cumulatively contribute to phospholipid signaling and three are known to impact gametogenesis. A recent preprint of wheat DGK2 genomic and expression profiles identified 20 genes and their upregulated expression in root tissues under salt and drought stress (Jia et al., 2020). The study did not sample grain tissue for transcriptome analysis. The larger TaDGK family likely masked a phenotypic response to loss of chromosome arm 5AS *TaDGK5A* in our HIF population and functional validation of TaDGK genes in wheat is an outstanding area of research.

Meiotic and mitotic cell divisions are elevated during flowering plant reproductive growth and organogenesis. Among the DEGs we identified *TraesCS5A02G110600*, an ortholog to Arabidopsis and rice subunit 6 of the augmin complex, which is responsible for microtubule nucleation during cell divisions (Hotta et al., 2012; Oh et al., 2016). Knockdown lines of *AUG6* in Arabidopsis disturbed mitotic and meiotic cell divisions as a result of malformed microtubule arrays and effected both male and female gamete development (Oh et al., 2016). Functional validation of genes that affect gamete development is challenging because of homozygous lethality. An alternative approach to studying the effect of *TraesCS5A02G110600* on HIF entries could be to measure cell size and number of developing grains, which has previously been associated with grain morphology variation in wheat (Brinton et al., 2017).

Given the dramatic genomic structural variation and relatively few variable visual phenotypes in the HIF population, there is likely more phenotypic variation than what meets the eye. For example, *TraesCS5A02G107800* orthologs *VTC2* and *VTC5* in Arabidopsis and *OsGGP* in rice catalyze the first step in the ascorbate biosynthetic pathway (Gao et al., 2011; Höller et al., 2015; Lim et al., 2016). Ascorbate is an essential signal‐transducing molecule for regular plant development, and loss‐of‐function *OsGGP* plants have significantly reduced biomass (80% loss), panicle number, and panicle weight. Knockout *VTC2* and *VTC5* plants and ascorbate deficiency have been associated with reduced growth by some studies, or more recently with an independent cryptic mutation (Gao et al., 2011; Lim et al., 2016). While we did not detect biomass variation on the order of magnitude measured in rice, hidden variation or homoeologous masking is likely at play. A future study of signal‐transducing molecule concentrations or hormone panels of the HIF population would likely unveil additional candidate genes.

### 4.4 Wheat positional cloning recommendations

Positional cloning in wheat has lagged behind the progress made in other staple crops in large part because of the redundant polyploid genome's masking of quantitative variation and the delayed availability of an annotated genome sequence. Even in an isogenic background, the missing chromosome 5A short arm was associated with only subtle phenotypic variation. Wheat deletion lines are classically associated with aberrant morphological variation or infertility, but our results demonstrate that large structural variation can go undetected for years across environments, research projects, and lab groups. Recent studies of 15 bread wheat cultivar genome assemblies showed that only 59% of each cultivar's genome was identical‐by‐state with other sequenced cultivars and detected extensive genomic rearrangements, underscoring the structural diversity of wheat (Brinton et al., 2020; Walkowiak et al., 2020). Our study illustrates that approaching positional cloning based on stable and robust biparental QTL mapping may overlook some hurdles unique to wheat's resilient polyploid genome. If one is going to invest the considerable resources necessary for successful positional cloning in wheat, or other polyploid crops, we propose the following recommendations:

1. **Move from a SNP to haplotype‐based approach to identify genetic diversity**. Genetic markers are often associated with a trait of interest but are not causal. In a fine‐mapping context this can overlook linkage, especially with broad genomic regions spanning the centromere, such as the GW 5A QTL, and result in selection of false positives (Platten et al., 2019). Haplotype blocks can identify genetic structural variation more comprehensively and precisely, as demonstrated by the recent release of 15 cultivar genome assemblies and accompanying visualization platform (Brinton et al., 2020; Walkowiak et al., 2020). Exome sequence‐capture data are available for far more wheat cultivars than genome assemblies, but safeguards need to be in place to detect and flag structural variation, for example in W7984 exome and regulatory‐capture (Gardiner et al., 2019; He et al., 2019a).
2. **Invest in sequencing to detect structural variants**. While the cost of whole‐genome sequencing in wheat is not yet feasible for individual breeding programs, long‐read sequencing and greater fold coverage (i.e., 10) has become increasingly affordable. Longer sequencing reads can detect even small‐ (30–10,000 bp) to mid‐scale (10,000–30,000 bp) structural variants, which impact trait diversity and are shown to be widespread in polyploid species (Chawla et al., 2021; Gabur et al., 2019; Mahmoud et al., 2019). For example, a recent study of *Brassica napus* L. found that up to 10% of all genes were affected by small‐ to mid‐scale structural variants including flowering‐time pathway genes, which can influence agronomic traits (Chawla et al., 2021). Obtaining long‐read sequencing (small‐ to mid‐scale structural variants) or 10‐fold coverage (large‐scale structural variants) of parental lines for a mapping population could become a prerequisite first‐step in positional cloning population development.
3. **Use the transcriptome to identify candidate genes**. RNA‐seq of isogenic material can identify differential expression and coding‐region allelic variation. This method alerted us to variants of interest arguably faster than pursuing additional HIF recombinants in the *QTgw.cnl‐5A* 10‐Mbp region. Measurements across multiple timepoints (i.e., >5) can open the door to developing gene networks for candidate gene discovery (Borrill et al., 2020). It is also notable that wheat transcriptome studies have reported large‐scale structural variants including interhomoeolog exchanges (He et al., 2017).
4. **Traits with broad overlapping QTL may not be pleiotropic**. The traditional yield components (spikes m^−2^, grain number per spike, and grain weight) are polygenic traits themselves. For example, in our study we showed that GL and GW can independently contribute to TGW. However, our hypothesis that a gene contributing to GW drove the TGW variation on chromosome arm 5AL overlooked the association with GW and chromosome arm 5AS. Eventually, in an isogenic background, we identified additive and hidden phenotypic variation associated with structural variation, rather than a single gene. Another recent example that has disrupted pleiotropic assumptions based on single‐marker trait associations was identified in a highly conserved region of chromosome 6A with a haplotype‐led approach (Brinton et al., 2020).

With an annotated reference genome and advancing gene editing techniques, positional cloning in wheat may become routine, but pinpointing quantitative phenotypic variation and causal genomic loci requires a more tactical approach in polyploids. Incorporating strategies that are sensitive to structural variation with classic positional cloning population development approaches will reduce the likelihood of mapping in the wrong direction. Fewer roadblocks to identifying a candidate gene will also lead to more efficient selection from TILLING populations or transgenic approaches during sequencing and functional validation (Krasileva et al., 2017). A comprehensive review of the latest advances of genomics and phenomics for trait discovery in polyploid wheat and gene functional characterization is given in Borrill et al. (2018) and Adamski et al. (2020)

## 5 CONCLUSION

The outcomes of this research challenge whether a causal gene variant approach to characterizing wheat grain yield components offers an efficient and sustainable path to genetic gains and food security. We set out to identify the causal variant underlying a previously characterized grain weight and morphology QTL, *QTgw.cnl‐5A*, using a well‐vetted, positional‐based cloning approach. We leveraged a HIF population, SNP genomic data, phenotypic associations, early grain development expression profiles, and predicted gene function to determine that *QTgw.cnl‐5A* was a result of strong LD with chromosome arm 5AS presence or absence (SynOpDH *r* ^2^ = 0.95, HIF *r* ^2^ = 0.91). Our results highlight that chromosome structural variation linkages can overpower the considerable resources required for positional cloning and that wheat harbors hidden phenotypic variation. Chromosome structural variation is common among polyploids, and the results and recommendations presented will be immensely useful for aiding future causal variant discovery. We also identified nine candidate genes on chromosome arm 5AS that may impact yield components; however, their practical application to breeding remains to be functionally validated. Given the resources required for individual gene validation, uncertain impact on final grain yield, and unknown response in a different genetic background, we argue that causal variant discovery for a complex quantitative trait like wheat yield requires an update to traditional positional‐based cloning approaches. Altogether, our findings demonstrate the phenotypic resiliency associated with polyploid genomic structural variation and provide recommendations for variant discovery strategies.

## AUTHOR CONTRIBUTIONS

Ella Taagen: Data curation; Formal analysis; Investigation; Methodology; Validation; Visualization; Writing‐original draft; Writing‐review & editing. James Tanaka: Project administration; Resources; Supervision; Writing‐review & editing. Alvina Gul: Data curation; Investigation; Methodology. Mark E. Sorrells: Conceptualization; Funding acquisition; Project administration; Supervision; Writing‐review & editing

## SUPPLEMENTAL MATERIAL

All of the data referenced in this manuscript, Supplemental files, and scripts for reproducing results are publicly available at: https://github.com/etaagen/Taagen_2021_TPG. Refer to .md files to view analysis output, and .Rmd files to view full script and reproduce results.

## CONFLICT OF INTEREST

The authors declare that there is no conflict of interest.
